# Neoadjuvant Stereotactic Radiotherapy for Brain Metastases: Systematic Review and Meta-Analysis of the Literature and Ongoing Clinical Trials

**DOI:** 10.3390/cancers14174328

**Published:** 2022-09-04

**Authors:** Paolo Palmisciano, Gianluca Ferini, Ramlah Khan, Othman Bin-Alamer, Giuseppe E. Umana, Kenny Yu, Aaron A. Cohen-Gadol, Tarek Y. El Ahmadieh, Ali S. Haider

**Affiliations:** 1 Department of Neurosurgery, University of Cincinnati College of Medicine, Cincinnati, OH 45220, USA; 2 Department of Radiation Oncology, REM Radioterapia srl, 95029 Viagrande, Italy; 3 College of Medicine, Texas A&M University, College Station, TX 77843, USA; 4 Department of Neurosurgery, University of Pittsburgh Medical Center, Pittsburgh, PA 15213, USA; 5 Department of Neurosurgery, Trauma Center, Gamma Knife Center, Cannizzaro Hospital, 95126 Catania, Italy; 6 Department of Neurosurgery, Memorial Sloan Kettering Cancer Center, New York, NY 10065, USA; 7 Department of Neurological Surgery, Indiana University School of Medicine, Indianapolis, IN 46290, USA; 8 Department of Neurosurgery, Loma Linda University Medical Center, Loma Linda, CA 92354, USA; 9 Department of Neurosurgery, The University of Texas M.D. Anderson Cancer Center, Houston, TX 77030, USA

**Keywords:** brain metastases, clinical trials, neoadjuvant radiotherapy, stereotactic radiosurgery, stereotactic radiotherapy

## Abstract

**Simple Summary:**

The available treatment strategies for patients with brain metastases remain suboptimal, with current research focused on identifying therapies intended to improve patient outcomes while reducing the risk of treatment-related complications. Several studies have investigated the role of pre-operative neoadjuvant stereotactic radiotherapy, and have proposed it as a valid alternative to post-operative adjuvant stereotactic radiotherapy. The aim of our systematic review was to comprehensively analyze the current literature and ongoing clinical trials evaluating neoadjuvant stereotactic radiotherapy in patients with brain metastases, describing treatment protocols and related outcomes. Early evidence suggests that neoadjuvant stereotactic radiotherapy may offer rates of local control and overall survival comparable to those obtained with adjuvant postoperative SRS, but comparative studies are currently lacking. In addition, neoadjuvant stereotactic radiotherapy shows low rates of post-treatment radiation necrosis and leptomeningeal metastases. Ongoing clinical trials aim to evaluate long-term outcomes in large patient cohorts, with some focused on comparing neoadjuvant stereotactic radiotherapy to adjuvant stereotactic radiosurgery.

**Abstract:**

Background: Brain metastases (BMs) carry a high morbidity and mortality burden. Neoadjuvant stereotactic radiotherapy (NaSRT) has shown promising results. We systematically reviewed the literature on NaSRT for BMs. Methods: PubMed, EMBASE, Scopus, Web-of-Science, Cochrane, and ClinicalTrial.gov were searched following the PRISMA guidelines to include studies and ongoing trials reporting NaSRT for BMs. Indications, protocols, and outcomes were analyzed using indirect random-effect meta-analyses. Results: We included 7 studies comprising 460 patients with 483 BMs, and 13 ongoing trials. Most BMs originated from non-small lung cell carcinoma (41.4%), breast cancer (18.7%) and melanoma (43.6%). Most patients had single-BM (69.8%) located supratentorial (77.8%). Patients were eligible if they had histologically-proven primary tumors and ≤4 synchronous BMs candidate for non-urgent surgery and radiation. Patients with primary tumors clinically responsive to radiotherapy, prior brain radiation, and leptomeningeal metastases were deemed non-eligible. Median planning target volume was 9.9 cm^3^ (range, 2.9–57.1), and NaSRT was delivered in 1-fraction (90.9%), 5-fraction (4.8%), or 3-fraction (4.3%), with a median biological effective dose of 39.6 Gy10 (range, 35.7–60). Most patients received piecemeal (76.3%) and gross-total (94%) resection after a median of 1-day (range, 1–10) post-NaSRT. Median follow-up was 19.2-months (range, 1–41.3). Actuarial post-treatment rates were 4% (95%CI: 2–6%) for symptomatic radiation necrosis, 15% (95%CI: 12–18%) and 47% (95%CI: 42–52%) for local and distant recurrences, 6% (95%CI: 3–8%) for leptomeningeal metastases, 81% (95%CI: 75–87%) and 59% (95%CI: 54–63%) for 1-year local tumor control and overall survival. Conclusion: NaSRT is effective and safe for BMs. Ongoing trials will provide high-level evidence on long-term post-treatment outcomes, further compared to adjuvant stereotactic radiotherapy.

## 1. Introduction

Brain metastases (BMs) are the most frequent intracranial tumors in adults, occurring in approximately 9–30% of patients with solid neoplasms, especially lung, breast, and melanoma [1,2]. Their incidence is constantly rising owing to the improved imaging surveillance and efficacy of systemic therapies in oncological patients, which favor longer survival but also tumor cell spreading to the central nervous system (CNS) due to limited blood-brain barrier (BBB) penetrance [3,4]. In view of their increased morbidity and mortality burden, the search for optimal management strategies with high effectiveness and reduced toxicity is of great interest [5]. Surgical resection remains key to provide tissue diagnosis and decompression with symptom relief, but results in local recurrence rates in up to 50% of cases [6]. Post-operative adjuvant radiotherapy, including whole brain radiotherapy (WBRT) and cavity boost stereotactic radiosurgery (SRS) or radiotherapy (SRT), proved to significantly improve local control (LC) and overall survival (OS) [7,8,9,10]. However, adjuvant WBRT carries high risks of cognitive deterioration and quality-of-life worsening, while adjuvant SRS has some challenges related to target delineation, patient logistics, and rates of post-treatment leptomeningeal metastases (LM) [10,11].

More recently, several studies proposed pre-operative neoadjuvant radiotherapy (NaSRT) approaches as valid alternatives to post-operative adjuvant radiotherapy protocols [12,13]. NaSRT protocols involve the pre-operative delivery of SRT to single or few (i.e., less than 3) BMs appropriate for resection, followed by surgical removal within the next 24–72 h [14,15]. Major rationales to prefer NaSRT over post-operative radiotherapy include improved tumor volume contouring and target delineation, shorter total treatment times, superior patient compliance, and reduced risks of radiation necrosis (RN) and post-surgery LM [15,16].

In patients with BMs, NaSRT protocols proved to be feasible and effective, achieving outcomes similar to post-operative SRT with lower complication rates. Yet, the limited number of studies and heterogeneity of most conclusions may raise some concerns for protocol generalizability. In this systematic review, we present available NaSRT protocols reported in the literature, meta-analyzing outcomes and survival. We further describe ongoing clinical trials, focusing on inclusion criteria and treatment protocols.

## 2. Materials and Methods

### 2.1. Literature Search

A systematic review was completed in accordance with the Preferred Reporting Items for Systematic Reviews and Meta-Analyses (PRISMA) guidelines (PROSPERO ID: CRD42022353017) [17]. PubMed, EMBASE, Scopus, Web of Science, and Cochrane were searched from database inception to April 16, 2022, using the search query: [(neoadjuvant OR pre-operative OR preoperative) AND (radiotherapy OR radiation OR radiosurgery) AND (brain metastases)]. Articles were exported to the reference manager software Mendeley (Elsevier, London, UK) and then deduplicated. A second search was performed in the same fashion on ClinicalTrial.gov to collect ongoing clinical trials.

### 2.2. Study Selection

A priori inclusion and exclusion criteria were defined. Studies written in English were included if they reported: (1) the use of NaSRT in single fraction (i.e., neoadjuvant stereotactic radiosurgery; NaSRS), or hypofractionated (NaSRT) protocols, (2) followed by planned surgical resection, (3) in patients with BMs, (4) with available data on management and outcomes. Studies were excluded if they were: (1) literature reviews, study protocols, or book chapters; (2) studies involving patients undergoing salvage surgical resection (i.e., not planned at the time of SRT) for clinical and/or imaging progression of BMs previously treated with radiotherapy; (3) studies involving patients receiving post-operative SRT. In case of studies involving identical cohorts of patients receiving NaSRT at the same institution, only the most recent were included.

Titles and abstracts of all collected articles were reviewed by two independent authors (P.P. and G.F.), who then screened full texts of studies that met the inclusion criteria. A third author (A.S.H.) solved any disagreements. Eligible articles were included upon the predefined criteria and references were searched to retrieve additional relevant studies.

### 2.3. Data Extraction

Data were extracted by one author (R.K.) and then confirmed independently by two additional authors (P.P. and A.S.H.). Missing data were not reported by the authors. Extracted data from published studies included: authors, year, cohort size, age, gender, primary tumor, per-patient number of BMs and location, indications for NaSRT, planning target volume (PTV), NaSRT protocol (i.e., prescribed dose and fractionation), type and extent of resection, follow-up, local and distant intracranial failure, local tumor control (LC), overall survival (OS), and survival status. Extent-of-resection was defined as “gross total resection” for 90–100% tumor resection and “subtotal resection” for 80–89% resection. Treatment outcomes were evaluated at the last available follow-up. The biologically effective doses (BED) were collected when available or calculated from raw data using an α/β ratio of 10 [18]. Data from ongoing trials were also extracted, including: trial number and institutions, design, estimated enrollment, eligibility criteria, intervention, experimental and/or comparator arm, primary and secondary outcomes. 

### 2.4. Data Synthesis and Quality Assessment

The primary outcomes of interest were treatment outcomes in patients with BMs undergoing NaSRT. Indications and management protocols were secondarily analyzed. The level of evidence of each article was evaluated upon the 2011 Oxford Centre For Evidence-Based Medicine guidelines [19]. Risk of bias was independently assessed for each article by two authors (P.P. and G.F.) using the Joanna Briggs Institute checklist [20]. The overall risk of bias of this review was determined by the aggregated risks of bias of all included studies.

### 2.5. Statistical Analysis

The software STATA 17.0 (StataCorp LLC, College Station, TX, USA) was used and bilateral *p*-values < 0.05 were considered significant for all statistical tests. Continuous variables are summarized as medians and ranges, while categorical variables as frequencies and percentages. Indirect meta-analyses were conducted for rates of radiation necrosis, local and distant failure, LM, LC, and OS. Pooled proportions of events (effect size, ES) were utilized to summarize outcomes, and the Wilson score method to compute confidence intervals (CI), both presented with forest plots [21]. The Freeman-Tukey transformation was operated to include studies with 0 or 1 event rate and to stabilize variance, and the DerSimonian and Laird approach for random effect models was applied to account for high between-studies variability [22,23]. Heterogeneity was assessed using the Higgins I-square (I2) and considered significant for I2 > 75% [24]. Publication bias was evaluated by detecting any evident visual asymmetry on generated funnel plots.

## 3. Results

### 3.1. Study Selection

**Figure 1:**
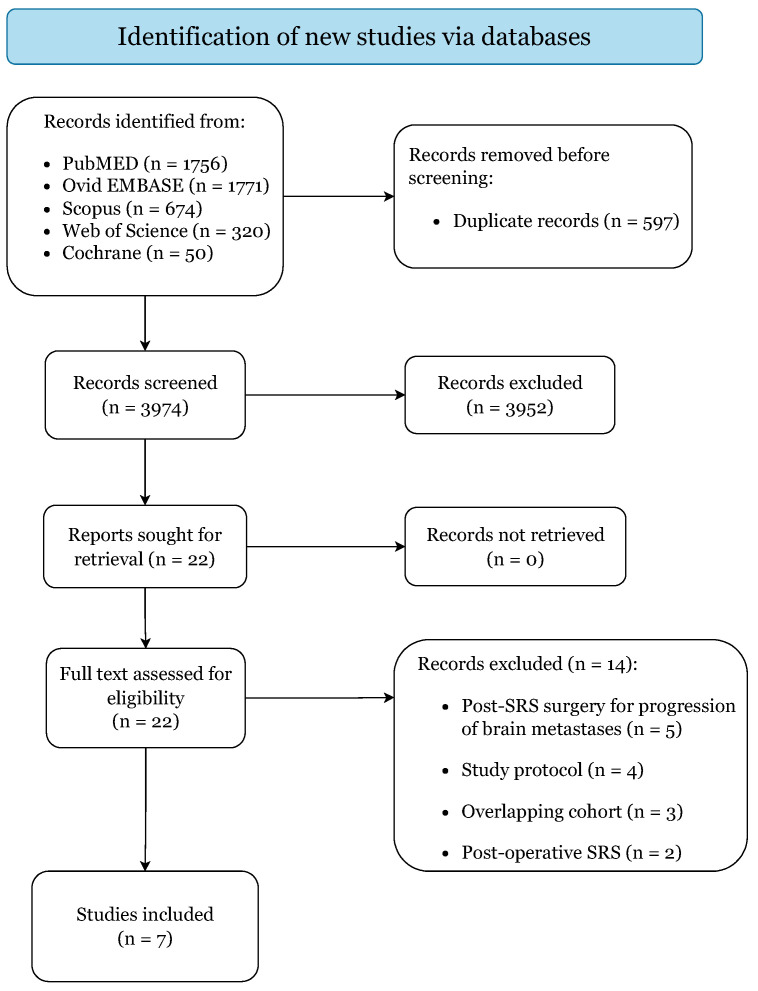
PRISMA 2020 Flow-Diagram.

Figure 1 illustrates the literature search and study selection process. The initial search yielded 4571 citations (PubMed: 1756; EMBASE: 1771; Scopus: 674; Web of Science: 320; Cochrane: 50). 

In accordance with the pre-specified criteria, 7 retrospective cohort studies were included, categorized as level IIb of evidence (Table 1) [25,26,27,28,29,30,31]. Quality assessment returned low risk of bias for all included articles, predisposing this study to an overall low risk of bias (Supplementary File S1). The studies from Asher et al. [12], Patel et al. [13], Patel et al. [32], and Prabhu et al. [33] were excluded because they included the same institutional cohort of patients published more recently in Prabhu et al. [27]. 

The second search returned 18 ongoing clinical trials, of which 13 met the inclusion criteria (Supplementary File S2) [34,35,36,37,38,39,40,41,42,43,44,45,46]. No evident visual asymmetry could be detected on the generated funnel plots, excluding the presence of publication bias (Supplementary File S3).

### 3.2. Demographics and Clinical Features

Table 2 summarizes the clinical characteristics of the 460 patients and the 483 treated lesions included in this review. Most patients were female (55%) enrolled to undergo NaSRT protocols at a median age of 60 years (range, 30–80). BMs mostly originated from primary non-small cell lung carcinoma (NSCLC; 41.4%), breast cancer (18.7%), and melanoma (14.6%). The majority of patients were treated for single BM (69.8%), most frequently supratentorial (77.8%). Only 15 (3.3%) and 7 (1.5%) patients were treated for synchronous 4 and ≥5 BMs, respectively [15,27,28].

### 3.3. Patient Eligibility and Treatment Protocols

All studies shared common eligibility criteria for NaSRT protocols, including patients with (1) 1–3 or 1–4 synchronous BMs, (2) from histologically-proven primary tumors, (3) requiring non-urgent resection for severe mass-effect symptoms or neurological deficits, (4) candidate to SRT. Patients were excluded if they: (1) had primary hematologic malignancy or radiosensitive tumor biology candidate to WBRT; (2) underwent prior WBRT and/or brain SRS/SRT; (3) required emergency decompressive surgery for life-threatening intracranial hypertension (with a preference towards adjuvant SRS/SRT protocols); (4) had evidence of pre-treatment LM. In addition, Patel et al. [26] included patients with symptomatic posterior fossa lesions and Deguchi et al. [29] excluded BMs eligible to be resected en bloc. 

The linear accelerator (LINAC) was used in the majority of cases (93.8%), with Prabhu et al. [28] also reporting the use of gamma knife (GK) and cyber knife (CK) in 23 (4.8%) and 7 (1.4%) patients, respectively. Median PTV was 9.9 cm3 (range, 2.9–57.1) (Table 2). In most studies, PTVs matched the gross total volumes (GTV) of target lesions calculated on contrast-MRI scans, while Kotecha et al. [30] and Udovicich et al. [31] added a margin of 1.5 mm and 1 mm to the delineated GTVs, respectively. Radiation was delivered at a median prescribed dose of 16.5 Gy (range, 12.6–35) and mostly with NaSRS protocols in 1-fraction (90.9%), followed by NaSRT protocols in 5-fractions (4.8%), and in 3-fractions (4.3%). The median calculated BED was 39.6 Gy10 (range, 35.7–60). Surgery was performed at a median of 1 day after NaSRT (range, 1–10). BM resection was completed in a piecemeal fashion in most cases (76.3%), achieving GTR (94%) more frequently than STR (6%).

### 3.4. Outcomes, Complications, and Survival

**Figure 2:**
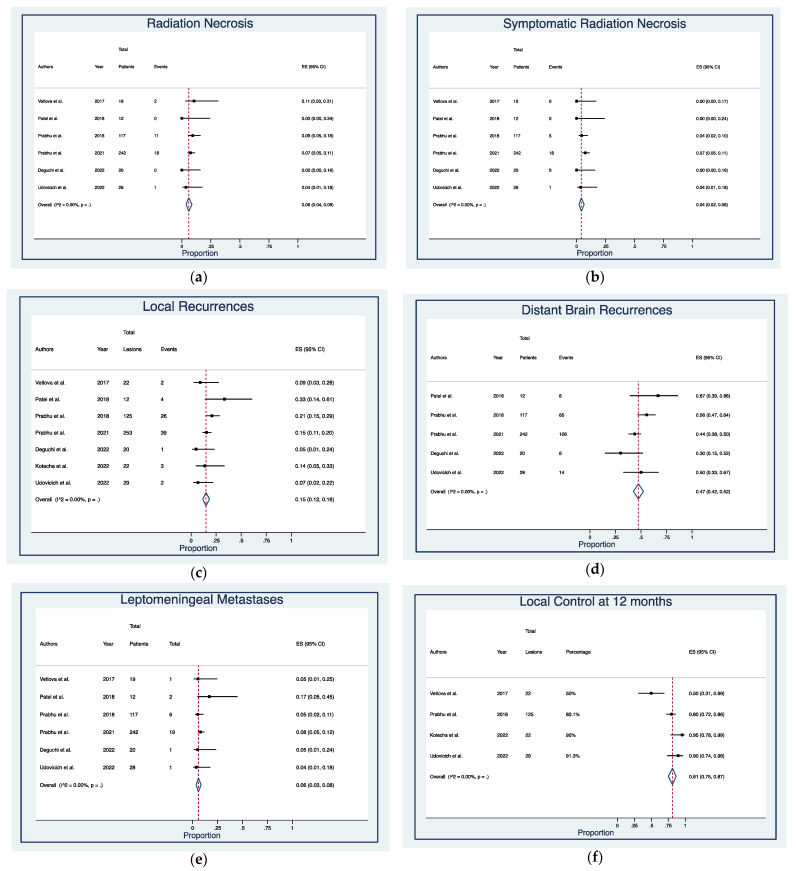

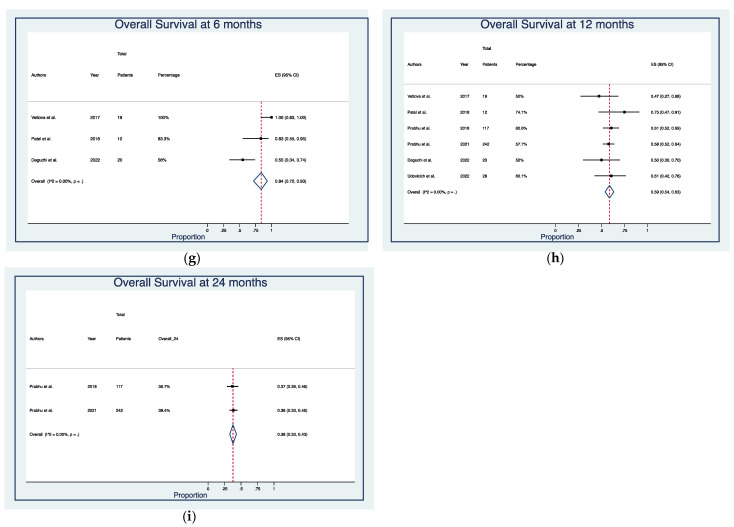
Forest plots of rates of (**a**) radiation necrosis [21,25,26,28,29,31], (**b**) symptomatic radiation necrosis [21,25,26,28,29,31], (**c**) local recurrences [25,26,27,28,29,30,31], (**d**) distant brain recurrences [26,27,28,29,31], (**e**) leptomeningeal metastases [25,26,27,28,29,31], (**f**) local control at 12 months [25,27,30,31], and overall survival at (**g**) 6 months [25,26,29], (**h**) 12 months [25,26,27,28,29,31], and (**i**) 24 months [27,28]. Squares define the proportions (effect size, ES) of individual studies and horizontal lines mark the 95% confidence intervals (CI). Diamonds indicate the pooled ES with 95% CI using the random effect model meta-analyses.

Median follow-up time was 19.2 months (range, 1–41.3) (Table 2). Post-treatment RN was detected in 32 cases (7.3%), of which 24 (5.5%) were symptomatic, with actuarial rates of 6% (95% CI: 4–9%) and 4% (95% CI: 2–6%), respectively (Figure 2a,b). Local and distant intracranial recurrences occurred in 77 (16.7%) and 199 (43.3%) patients, with actuarial rates of 15% (95% CI: 12–18%) and 47% (95% CI: 42–52%) (Figure 2c,d). LM was diagnosed at imaging and/or cerebrospinal fluid (CSF) analysis in 30 patients (6.8%), with actuarial rates of 6% (95% CI: 3–8%) (Figure 2e). Pooled 1-year LC rates were 81% (95% CI: 75–87%) (Figure 2f). Most patients were dead at last follow-up (66.7%), with pooled OS rates of 84% (95% CI: 72–93%) at 6-months, 59% (95% CI: 54–63%) at 1-year, and 38% (95% CI: 33–43%) at 2-year (Figure 2g–i)). 

### 3.5. Ongoing Clinical Trials: Eligibility Criteria, Protocols, and Outcome Measures

Thirteen interventional clinical trials are currently ongoing: 8 single group [35,38,39,40,41,42,43,45], 4 randomized [34,36,37,46], and 1 non-randomized [44] (Supplementary File S2). Eligibility criteria are largely shared across all ongoing trials, enrolling patients with: (1) age ≥ 18 years; (2) favorable baseline performance status (Karnofsky ≥ 60–70 or Eastern Cooperative Oncology Group 0–2); (3) histological diagnosis of primary tumors; (4) no contraindications to MRI; (5) ≤3–6 contrast-enhancing BMs with maximum diameter ≥1 cm and ≤4–6 cm, one indicated for surgical resection; (6) eligibility for SRS or SRT; (7) estimated survival ≥ 3–12 months; (8) negative pregnancy test or contraceptive medications; (9) ability to complete neurocognitive assessment and provide informed written consensus. Patients are excluded if they have: (1) radiosensitive tumor histology (e.g., leukemia, lymphoma, germ cell tumors, small cell lung carcinoma, brain tumors) candidate to WBRT or radiation only protocols; (2) BMs causing ≥ 5–10 mm midline brain shift, 4th ventricle compression, and/or severe intracranial hypertension requiring emergency decompressive surgery; (3) BMs close to the optic pathway and/or brainstem; (4) imaging and/or CSF-diagnosed LM; (5) a history of prior WBRT and/or SRS/SRT to the lesion to be resected; (6) a history of prior cytotoxic chemotherapy and/or anti-VEGFR therapy; (7) psychological disorders, unstable illnesses, or other personal reasons likely to interfere with compliance to treatment and follow-ups. In most trials, treatment protocols consist of NaSRS with consequent BM resection, except for the ones led by Brun [35] and Wu [38], which involve the use of hypofractionated NaSRT. The 4 randomized clinical trials are expected to compare patients undergoing NaSRS versus patients receiving post-surgery SRS/SRT [34,36,37,46]. The non-randomized clinical trial led by Buchwald [44] is expected to compare the use of high-dose versus low-dose steroid therapy in patients undergoing NaSRS. The most common primary outcome measures across all trials comprise LC and LM rates at 6-month and 1-year, followed by radiation toxicity rates and/or maximal tolerated doses. Of note, the trial led by Bovi [39] is intended to evaluate the cured rate at 20-month, and the trial led by Yan [46] is expected to assess the CNS composite endpoint event at 60-month. The most common secondary outcomes of interest are rates of LC, distant failure, OS, radiation toxicity, and LM at 3-month to 2-year follow-ups. Some trials will also analyze the post-treatment cognitive status and quality-of-life of enrolled patients [34,35,36,37,43,46], while the trial led by Agrawal [40] will evaluate the correlation between RNA biomarkers and LC at 12-month and the trial led by Murphy [45] will assess the rate of patients requiring salvage therapy at 36-month.

## 4. Discussion

Starting from the study of Asher et al. published in 2014 [12], the interest and use of NaSRT for BMs has gradually expanded, with a constantly growing number of published studies and ongoing trials led by multiple institutions worldwide. As per the initial findings, further confirmed by this systematic review, NaSRT protocols offer high rates of LC while minimizing the risks of symptomatic RN and LM, which represent two of the direst complications in patients receiving adjuvant SRS/SRT after BM resection [13]. Several ongoing clinical trials are currently conducted to investigate the long-term outcomes of NaSRT in terms of LC and LM-free rates, especially in comparison to post-surgical adjuvant SRS/SRT protocols.

WBRT has been historically used for the palliative management of patients with BMs [47], with surgical resection planned in cases of large lesions exerting considerable mass effect on the surrounding brain and/or necessitating histological diagnosis [48]. Later, randomized clinical trials observed that surgery prior to WBRT correlated with significant OS improvement compared to WBRT alone [49], and with significantly lower rates of intracranial failure than surgery alone [6]. Owing to the adverse events related to surgery, namely brain tissue manipulation with injury to the functional cortex and white matter tracts, and WBRT, namely neurotoxicity with neurocognitive impairment, SRS gained increasing interest as stand-alone or adjunct therapy for 1–3 BMs [50,51,52]. As large BMs (>3 cm) causing intracranial hypertension with neurological deficits requiring surgery, adjuvant SRS to the postoperative cavity has been largely investigated, with 1-year LC rates ranging 70–90% and variable OS achieved across several retrospective cohorts [53]. Two randomized trials confirmed that postoperative SRS correlated with higher LC rates but also superior risks of LM compared to surgery alone [9], and with lower neurocognitive deficits but also inferior distant intracranial control compared to postoperative WBRT [8]. No differences in OS were found in both trials. Newer radiation techniques, including intracavitary brachytherapy and intraoperative radiotherapy, have been recently introduced, mainly for the management of recurrent BMs. However, they necessitate further larger investigations before being implemented as gold-standard therapies [54,55]. Moreover, some challenging bulky asymptomatic BMs may also benefit from neoadjuvant or definitive spatially fractionated radiation techniques, which allow for the delivery of non-homogeneously large stereotactic doses while avoiding concerns about an alarming dose-volume effect [56,57]. The SRT’s dose distribution may be adapted to the non-homogeneity of tumor oxygenation, intended to overcome the radioresistant tumor hypoxic sub-volumes while eliciting a useful bystander effect on the under-dosed areas [58,59]. However, at the present time, the usefulness of this approach remains only theoretical, as the current imaging techniques are not able to correctly detect the tumor oxygen landscape. In addition, the knowledge on the treatment-related adverse events in such a critical body area is still lacking [60].

The perceived drawbacks related to postoperative adjuvant SRS led to the idea of implementing NaSRS protocols before BM resection. This is in line with the recent guidelines for breast and gastrointestinal cancers that advocate neoadjuvant radiotherapy as the standard of care [61,62]. The logistics and planning benefits of NaSRT over postoperative SRS have been largely discussed across our included articles. NaSRT protocols allow for improved accuracy in target delineation of intact brain metastases for pre-planning contouring, as BMs’ contrast-enhancing borders are readily identifiable and easily distinguished from the normal brain tissue [15,28]. In contrast, the surgical manipulation leads to major postoperative changes within the surgical field, which increase the interobserver variability in delineating BM’s borders for adjuvant radiotherapy [63]. This results in different PTV definition between the two modalities: while for adjuvant SRS protocols a margin of 1–2 mm is generally required to be added to the postoperative cavity to reduce the risk of geographical miss, for NaSRT protocols the PTV matches the GTV in most cases, with no required margins to be added [27,32]. In addition, a recent international consensus advocated the delivery of postoperative radiation also to the resection corridor targeting tumor cells potentially translocated during the operation [64], not required for NaSRT. Hence, lower volumes of normal brain tissue are likely to be exposed to radiation in NaSRT settings, likely correlating with reduced risks of surgical wound dehiscence and RN, which is dose-dependent other than volume-dependent [26,31,65]. Higher patient compliance is also expected with NaSRT, as patients undergo radiation and surgery during the same hospitalization, with a reduced time burden and costs compared to adjuvant SRS [28]. Adjuvant SRS is usually performed after 2–5 weeks to reduce the degree of postoperative changes when contouring PTVs and the patient’s discomfort when applying the frame close to the surgical wound [16]. In addition, delayed post-surgery radiation has been also shown to increase the risk of intracranial recurrences [9]. From a radiobiological perspective, radiotherapy induces the tumor’s DNA damage by generating oxygen-based free radicals from oxygen molecules supplying the tumor [66]. While adjuvant SRS delivers radiation to hypoxic postoperative beds, NaSRT delivers radiation to tumors with intact blood supply and oxygenation, thus requiring lower doses to achieve similar control of microscopic residual. This was confirmed by the 20% dose reduction implemented across all included studies [27,28]. NaSRT is also expected to reduce the risk of post-surgery tumor spillage and LM, especially with piecemeal resection, by pre-treating cancer cells fated for intraoperative transposition and seeding with the CSF [29,53]. Despite the envisioned advantages, potential pitfalls of NaSRT approaches should also be noted, namely the lack of histological confirmation before starting radiation protocols. This may lead to overtreating patients with different diagnoses not requiring radiotherapy and dynamic changes in treatment planning during the course of treatment, which may cause not completion of BM resection [15]. In addition, Prabhu et al. [28] reported a possible increased risk of surgical wound complications after NaSRT, noted in 3 patients (1.2%) of their multi-institutional cohort. Although their incidence was not sufficient enough to confirm any association between NaSRT and surgical wound dehiscence, the authors suggested that higher radiation doses coupled with BM’s bony infiltration should be considered as risk factors for postoperative wound complications and, thus, should be managed with special care [28].

The eligibility criteria for NaSRT are somewhat similar to those for adjuvant SRS, noted to be mostly shared across all the published studies and ongoing clinical trials [14]. Adult patients with BMs are deemed candidates for NaSRT if they were histologically-diagnosed with primary solid tumors, not preferred to be treated only with radiotherapy (contrarily to radiosensitive tumors such as germ cell carcinomas) [31]. Based on the SRS’s best action against few and small intracranial lesions, most studies selected only patients with ≤3 synchronous BMs with ≤5 cm maximal diameters, and requiring surgical resection as a result of symptomatic mass effect with neurological deficits [28]. Contrarily, patients were excluded when reporting a history of prior WBRT or SRS/SRT delivered to the same targeted lesion, requiring emergency decompressive surgery due to BM-related life-threatening intracranial hypertension, or being diagnosed with disseminated LM [27]. Of note, as Deguchi et al. [29] mainly focused on evaluating the role of NaSRT after piecemeal BM resection, the authors included only lesions deemed non-eligible to be resected en bloc. Treatment protocols were also largely similar among included studies, frequently characterized by single-fraction NaSRS sessions followed within 24–48 h by BM removal mostly performed in a piecemeal fashion and intended to achieve GTR [25,28,30]. The only exceptions were the studies of Deguchi et al. [29] and Udovicich et al. [31], which described the use of 3-fraction NaSRT (also called hypofractionated SRS) and 5-fraction NaSRT, completed 1 to 5 days before BM resection. Similarly to adjuvant SRS, hypofractionated and single-fraction NaSRS are both favorable for small-sized lesions (< 3–5 cm^3^). Despite the limited available data on hypofractionated adjuvant SRS compared to standard single-session adjuvant SRS for BMs, previous studies have suggested that hypofractionated SRS may correlate with lower risks of radiation-related complications to the healthy brain tissue surrounding the targeted lesions [50,51,52]. However, a pitfall of performing hypofractionated NaSRS compared to single-session NaSRS comprises the requirement to have the patients undergo more than one radiotherapy session. This may prove to be particularly challenging in patients with high levels of anxiety or with personal or social difficulties to reach the treatment centers. A more in-depth analysis of the clinical benefits of hypofractionated versus single-session SRS approaches may be required also within the field of NaSRT, to better define individual patients’ and tumors’ characteristics that may suggest the benefit to perform one approach or the other on a case-by-case basis. Multi-session NaSRT protocols correlated with higher BEDs than single-session NaSRS, which were likely responsible for the higher rates of LC but also of RN and LM. Although these findings have been obtained only in small retrospective cohort studies and require external validation, single-session NaSRS may still be preferred over multi-session NaSRT due to the comparable OS rates and the likely lower impact on a patient’s quality of life.

The pooled results obtained from our collected studies have comprehensively confirmed the safety and efficacy of NaSRT protocols for patients with BMs. NaSRT appears to be mostly effective in achieving favorable LC (1-year actuarial rates 81%) while minimizing the risk of RN (actuarial rates 6%), especially treatment-requiring symptomatic RN (actuarial rates 4%), and LM (actuarial rates 6%). However, less favorable pooled outcomes were obtained for distant recurrences (actuarial rates 47%) and OS (actuarial rates 84%, 59%, and 38% at 6-month, 1-year, and 2-year, respectively). We assume that these findings are likely related to the mechanism of action of SRT, which selectively target restricted volumes achieving optimal local tumor control with minimal radiation toxicity to the healthy brain tissue, but also are insufficient to treat distant tumor cells and alter the systemic disease course. NaSRS-related outcomes have been further compared by Patel et al. [13] and Prabhu et al. [33] to those obtained in BMs after adjuvant SRS. The authors found no significant differences in LC between the two modalities, but significantly lower rates of RN and LM in patients receiving NaSRS. A third study from Patel et al. [32] also compared NaSRS to postoperative WBRT, observing no significant differences in rates of RN, LC, distant failure, and OS between the two cohorts but a significantly lower incidence of LM in patients treated with WBRT. Yet, as these results have been obtained from heterogeneous and retrospective cohorts of patients, they are still required to be validated with larger, prospective, and multi-institutional studies.

Our systematic search identified 13 ongoing interventional clinical trials investigating the role of NaSRT in BMs. The eligibility criteria and protocols have been devised in accordance with the studies already published within the literature. The goal was to collect homogenous cohorts of patients with no major contraindications to NaSRT and who were not candidates to different treatment approaches, such as urgent surgery or stand-alone radiotherapy. Such finding highlights the expected high clinical relevance of this modality, with all trials intended to provide long term efficacy and safety outcomes that will assist the definition of future guidelines for the multidisciplinary radiotherapy management of patients with BMs. The current trend in the treatment of BMs is focused on devising patient-tailored, tumor-specific, and minimally invasive approaches that may offer good and prolonged tumor control while limiting any risk of treatment-related complications. Based on the available findings provided by the current literature and the strong clinical interests in the new ongoing trials, NaSRT is likely to offer promising therapeutic options for patients with BMs, but it should be evaluated within the context of tumor-tailored systemic therapies. In view of the current development of next generation sequencing also for BMs, the ongoing trials should also investigate the efficacy and risks of NaSRT, in combination with patient-specific targetable therapies and immunotherapies matching specific mutations identified after molecular testing from systemic tissue or blood. Of note, 4 randomized controlled trials are currently enrolling BM patients for comparing post-radiation outcomes between NaSRS and adjuvant SRS protocols [34,36,37,46]. Although these results will be available in a few years, these trials will provide the highest level of evidence on the impact of NaSRT in patients with BMs, and, potentially, change their current standard of care.

### Limitations

Our review has some limitations. All included studies were retrospective, likely to be exposed to selection bias, and published only from a few US institutions and one Russian institution. Owing to the limited and overlapping data comparing NaSRT with adjuvant SRS, we could not perform comparative meta-analyses between the two modalities. The limited granular data on performance status scores, neurocognitive status, complications, salvage therapy, and adjuvant systemic therapies, prevented further outcome analyses.

## 5. Conclusions

Early evidence suggests that NaSRT is feasible and safe for the management of selected patients with BMs. Reported rates of LC and OS are overall comparable to those obtained with adjuvant postoperative SRS, but comparative studies are currently lacking. The major expected benefits of NaSRT appear to be related to its low rates of post-treatment RN and LMs, suggesting its promising role in patients with high risks of LM dissemination. Yet, the restricted indications and protocols may limit its implementation in patients presenting with multiple and large BMs requiring early neurosurgical treatment and/or previously treated with radiotherapy. Ongoing clinical trials have been set to evaluate long-term outcomes, mainly LC and neurotoxicity in large patient cohorts, with some focused on comparing NaSRT to adjuvant SRS to guide the definition of the best standards of care.

## Figures and Tables

**Table 1:** Overview of all included studies.

Authors—Year	Patients/ Lesions	Planning Target Volume cm^3^ Median (Range)	Dose (Gy) & Fraction (fr) Median (Range)	Extent of Resection	Radiation Necrosis/Symptomatic	Local Failure/Distant Failure	Overall Survival
Vetlova, 2017 [25]	19/ 22	14.1 (3–57.1)	18 Gy (12.6–24.4) in 1 fr	GTR 22 (100%)	2 (10.5%)/ 0 (0%)	2 (10.5%)/ N/A	6 m 100% 1 y 50%
Patel, 2018 [26]	12/ 12	14.7 (3.4–34.8)	16 Gy (12–21) in 1 fr	GTR 12 (100%)	0 (0%)/ 0 (0%)	4 (33.3%)/ 8 (66.7%)	6 m 83.3% 1 y 74.1%
Prabhu, 2018 [27]	117/ 125	8.3 (4.6–13.3)	15 Gy (14–17) in 1 fr	GTR 119 (95.2%) STR 6 (4.8%)	11 (9.4%)/ 5 (4.3%)	26 (20.8%)/ 65 (55.6%)	1 y 60.6% 2 y 36.7%
Prabhu, 2021 [28]	242/ 253	9.9 (5–17)	15 Gy (14–16) in 1 fr (1–5)	GTR 237 (93.7%)/ STR 16 (6.3%)	18 (7.1%)/ 18 (7.1%)	15 Gy (14–16) in 1 fr (1–5)	39 (15.4%)/ 106 (43.8%)
Deguchi, 2022 [29]	20/ 20	17.6 (5.6–49.7)	30 Gy (30–35) in 5 fr	GTR 17 (85%) STR 3 (15%)	0 (0%)/ 0 (0%)	1 (5%)/ 6 (30%)	6 m 56% 1 y 50%
Kotecha, 2022 [30]	22/ 22	14.2 (2.9–31.4)	18 Gy (15–30) in 1 fr (1–5)	GTR 22 (100%)	N/A	3 (1.6%)/ N/A	N/A
Udovicich, 2014 [31]	28/ 29	4.5 (3.1–18.9)	23 Gy (18–27.5) in 3 fr (1–5)	GTR 25 (86.2%) STR 4 (13.8%)	1 (3.4%)/ 1 (3.4%)	2 (7.1%)/ 14 (50%)	1 y 60.1%

**Table 2:** Summary of clinical characteristics, treatment protocols, and pooled outcomes.

Characteristics	Value
Cohort size (no.)	
Patients	460
Lesions	483
Demographics	
Age (years), median (range)	60 (30–80)
Gender (female)	253 (55%)
Primary Tumor	No. (%)
Non-small cell lung carcinoma	190 (41.4%)
Breast cancer	86 (18.7%)
Melanoma	67 (14.6%)
Renal cell carcinoma	43 (9.3%)
Others	74 (16.1%)
Number of Lesions Per-Patient	No. (%)
1	321 (69.8%)
2	79 (17.2%)
3	38 (8.3%)
4	15 (3.3%)
≥5	7 (1.5%)
Location	No. (%)
Supratentorial	358 (77.8%)
Infratentorial	102 (22.2%)
Planning Target Volume (cm^3^), median (range)	9.9 (2.9–57.1)
Radiotherapy Protocol	
Prescribed dose (Gy)	16.5 (12.6–35)
Number of fractions	
1	439 (7.5%)
3	21 (6.9%)
5	23 (4.9%)
Biologically effective dose (BED) (Gy_10_)	39.6 (35.7–60)
Time from Radiotherapy to Surgery (day), median (range)	1 (1–10)
Surgery Protocol	No. (%)
Type of resection	
Piecemeal	180 (76.3%)
En bloc	56 (23.7%)
Extent of resection	
Gross-total	454 (94%)
Subtotal	29 (6%)
Follow-up (months), median (range)	19.2 (1–41.3)
Outcomes	No. (%)
Radiation necrosis (*n* = 438)	32 (7.3%)
Symptomatic	24 (5.5%)
Local recurrences	77 (16.7%)
Distant brain recurrences (*n* = 419)	199 (43.3%)
Leptomeningeal metastases (*n* = 438)	30 (6.8%)
Local tumor control (*n* = 186)	
1-year	80% (50–95%)
Overall survival (*n* = 438)	
6-month	80% (56–100%)
1-year	58% (50–74.1%)
2-year	37.8% (36.7–38.4%)
Survival Status (*n* = 438)	No. (%)
Alive	146 (33.3%)
Dead	292 (66.7%)

## Supplementary Materials

The following are available online at https://www.mdpi.com/article/10.3390/cancers14174328/s1, Supplementary File S1: Risk of bias assessments for all included studies. Supplementary File S2. Overview of all ongoing clinical trials [34,35,36,37,38,39,40,41,42,43,44,45,46]. Supplementary File S3. Funnel plots of pooled rates of: A. radiation necrosis; B. symptomatic radiation necrosis; C. local recurrences; D. distant brain recurrences; E. leptomeningeal disease; F. local control at 12 months; G. overall survival at 6 months; H. overall survival at 12 months; I. overall survival at 24 months.
