# Supplementary material for: Neoadjuvant Stereotactic Radiotherapy for Brain Metastases: Systematic Review and Meta-Analysis of the Literature and Ongoing Clinical Trials
Source: Cancers (Basel). 2022 Sep 4;14(17):4328. doi: 10.3390/cancers14174328 (PMC9455064; doi:10.3390/cancers14174328)
Supplement: Supplementary file 1 [file cancers-14-04328-s001.zip › Supplementary File S1 - Risk of bias.pdf]

Supplementary File S1. Risk of bias assessments for included studies.

**Joanna Briggs Institute Checklist – Criteria**
1. Were there clear criteria for inclusion in the case series?
2. Was the condition measured in a standard, reliable way for all participants included in the case series?
3. Were valid methods used for identification of the condition for all participants included in the case series?
4. Did the case series have consecutive inclusion of participants?
5. Did the case series have complete inclusion of participants?
6. Was there clear reporting of the demographics of the participants in the study?
7. Was there clear reporting of clinical information of the participants?
8. Were the outcomes or follow up results of cases clearly reported?
9. Was there clear reporting of the presenting site(s)/clinic(s) demographic information?
10. Was statistical analysis appropriate?
**Responses Options**: Yes, No, Unclear, Not Applicable (NA)
**Quality Rating**: Poor 0 – 3; Fair 4 – 7; Good 8 – 10

Study	1	2	3	4	5	6	7	8	9	10	Rating
Vetlova et al. – 2017[25]	Yes	Yes	Yes	Yes	Yes	Yes	Yes	Yes	Yes	No	9 – Good
Patel et al. – 2018[26]	Yes	Yes	Yes	Yes	Yes	Yes	Yes	Yes	Yes	Yes	10 – Good
Prabhu et al. – 2018[27]	Yes	Yes	Yes	Yes	Yes	Yes	Yes	Yes	Yes	Yes	10 – Good
Prabhu et al. – 2021[28]	Yes	Yes	Yes	Yes	Yes	Yes	Yes	Yes	Yes	Yes	10 – Good
Deguchi et al. – 2022[29]	Yes	Yes	Yes	Yes	Yes	Yes	Yes	Yes	No	NA	8 – Good
Kotecha et al. – 2022[30]	Yes	Yes	Yes	Yes	Yes	Yes	Yes	Yes	Yes	Yes	10 – Good
Udovicich et al. – 2022[31]	Yes	Yes	Yes	Yes	Yes	Yes	Yes	Yes	Yes	NA	9 – Good
