# Supplementary material for: Neoadjuvant Stereotactic Radiotherapy for Brain Metastases: Systematic Review and Meta-Analysis of the Literature and Ongoing Clinical Trials
Source: Cancers (Basel). 2022 Sep 4;14(17):4328. doi: 10.3390/cancers14174328 (PMC9455064; doi:10.3390/cancers14174328)
Supplement: Supplementary file 1 [file cancers-14-04328-s001.zip › Supplementary File S2 - Overview trials.pdf]

Supplementary File S2. Overview of all ongoing clinical trials.

Principal Investigators (Trial Number)	Study Type – Phase	Institution(s)	Estimated enrollment	Inclusion criteria	Exclusion criteria	Intervention	Comparator Arm	Primary outcomes	Secondary outcomes
**Rogers [34] (NCT05124236)**	Interventional – II Randomized, Parallel assignment	Kantonsspital Aarau	200	1) Age ≥18 years; 2) KPS ≥60; 3) Histological diagnosis primary tumor; 4) Able take steroids; 5) No contraindication MRI; 6) MRI-diagnosed ≤3 contrast-enhancing brain metastases of ≤4cm diameter; 7) estimated survival >12 months; 8) PLT >100/mL, INR <1.3, Hb >7.5g/dL; 9) Willing and able to give consent and participate in all evaluations	1) Radiosensitive tumor histology (germ cell, lymphoma, multiple myeloma); 2) >10mm midline shift, 4th ventricle compression, signs intracranial hypertension requiring emergency decompression; 3) ≥4 brain metastases and/or ≥4cm diameter; 4) >1 metastases requiring resection; 5) LM in CSF or at MRI; 6) prior WBRT or SRS/SRT to the lesion to be resected; 7) prior non-meningioma tumor diagnosis and/or resection; 8) prior radionucleide therapy within 30 days; 9) prior anti-VEGF therapy within 6 weeks; 10) pregnancy, lactation, or not willing to undergo contraception	NaSRS + Tumor resection	Tumor resection + Postoperative hfSRT	1) LM rate at 12-month	1) Local control at 3/6/12-month; 2) Distant failure at 3/6/12-month; 3) Radiation necrosis at 3/6/12-month; 4) Quality of life (QLQ30 and BN20) at 3/6/12-month
**Brun [35] (NCT04503772)**	Interventional – II Single group	Group Interregional de Recherche Clinique et d'innovation	70	1) Age ≥18 years; 2) KPS ≥70; 3) Histological diagnosis primary tumor; 4) Affiliation to the French social security system; 5) No contraindication MRI; 6) MRI-diagnosed ≤4 brain metastases of ≤5cm diameter, one indicated for surgery; 7) estimated survival >6 months; 8) Negative pregnancy test ≥7 days prior to start SRS; 9) Willing and able to give consent and participate in all evaluations	1) Radiosensitive tumor histology (germ cell, lymphoma, multiple myeloma, leukemia); 2) Metastases from SCLC, renal cancer, melanoma, sarcoma; 3) >5mm midline shift, 4th ventricle compression, signs intracranial hypertension requiring emergency decompression; 4) >4 brain metastases; 5) LM in CSF or at MRI; 6) prior WBRT or SRS/SRT to the lesion to be resected; 7) proximity to organs at risk not allowing prescribed target dose; 8) surgical delay >3days after SRS; 9) prior anti-VEGF therapy within 6 weeks; 10) pregnancy, lactation, or not willing to undergo contraception; 11) Psychological disorder or other reasons interfering with patient compliance	HfNaSRS + Tumor resection	No	1) Local control at 6-month	1) Local control at 12-month; 2) Distant control at 3/6/9/12-month; 3) Radiation necrosis at 12-month; 4) Overall survival at 3/6/9/12-month; 5) Acute (<3-month) and delayed (>3-month) toxicities; 6) Predictive factors; 7) Cognitive function (MMSE) at 3/6/9/12-month; 8) Quality of life (QLQ30) at 3/6/9/12-month
**Bovi [39] (NCT04545814)**	Interventional – II Single group	Froedtert Hospital & Medical College of Wisconsin	10	1) Age ≥18 years; 2) SRS candidate; 3) MRI-diagnosed ≤4 contras-enhancing brain metastases of ≤5cm diameter, one indicated for surgery; 4) estimated survival >6 months; 5) Surgical resection ≤10 days after SRS; 6) postmenopausal ≥1 year or contraception use; 7) Willing and able to give consent and participate in all evaluations	1) Planned adjuvant focal brain therapy; 2) ≥4 brain metastases and/or ≥5cm diameter; 3) LM in CSF or at MRI; 4) prior WBRT or SRS/SRT to the lesion to be resected; 5) prior anti-VEGF therapy within 6 weeks; 6) pregnancy, lactation, or not willing to undergo contraception; 7) Psychological disorder or other reasons interfering with patient compliance	NaSRS + Tumor resection	No	1) Cured rate at 20-month (no. patients with no identifiable disease at MRI)	1) Progression free survival at 6/12/18-month; 2) Overall survival at 6/12/18-month; 3) LM at 24-month; 4) Radiation necrosis at 24-month; 5) Quality of life (MDASI-BT) every 3 months up to 24-month
**Agrawal [40] (NCT03398694)**	Interventional – II Single group	Indiana University School of Medicine	50	1) Age ≥18 years; 2) Resection ≤4 days after SRS; 3) No contraindication MRI; 4) MRI-diagnosed ≤4 brain metastases of ≤5cm diameter; 5) estimated survival	1) >4 brain metastases and/or ≥5cm diameter; 2) LM in CSF or at MRI; 3) prior WBRT or SRS/SRT to the lesion to be resected; 4) prior anti-VEGF therapy within 6 weeks; 5) pregnancy, lactation, or not willing to undergo	NaSRS + Tumor resection	No	1) Local control at 6-month	1) Overall survival at 6/12/24-month; 2) Progression free survival at 6/12/24-month; 3) Distant failure at 24-

				>6 months; 6) PLT >100/mL, INR <1.3, Hb >7.5g/dL, ANC>1500/count; 7) Willing and able to give consent and participate in all evaluations	contraception; 7) Psychological disorder or other reasons interfering with patient compliance				month; 4) Radiation necrosis at 24-month; 5) LMD at 24-month; 6) Correlation with RNA biomarkers
**Shultz [41] (NCT03368625)**	Interventional – II Single group	University Health Network, Toronto	30	1) Age ≥18 years; 2) KPS ≥60; 3) No contraindication MRI; 4) MRI-diagnosed ≤6 contrast-enhancing brain metastases of >2cm and <4cm diameter; 5) estimated survival ≥3 months; 6) Willing and able to give consent and participate in all evaluations	1) Radiosensitive tumor histology (germ cell, lymphoma, SCLC, seminoma, primary brain tumor); 2) lesion ≤2mm close to the optic chiasm; 3) midline shift, 4th ventricle compression, signs intracranial hypertension requiring emergency decompression; 4) LM in CSF or at MRI; 5) prior WBRT or SRS/SRT to the lesion to be resected; 6) prior chemotherapy within <7 days; 10) pregnancy, lactation, or not willing to undergo contraception	NaSRS + Tumor resection	No	1) Radiation toxicity at 12-month	1) Local control at 12-month; 2) Progression free survival at 12-month; 3) Overall survival at 12-month; 4) LM at 12-month
**Shiao [42] (NCT03163368)**	Interventional – II Single group	Cedars-Sinai Medical Center	25	1) Age ≥18 years; 2) KPS ≥60; 3) Histological diagnosis primary tumor; 4) MRI-diagnosed brain metastases of <4cm diameter; 5) estimated survival >3 months; 6) Negative pregnancy test ≥7 days prior to start SRS; 7) Willing and able to give consent and participate in all evaluations	1) Radiosensitive tumor histology (germ cell, lymphoma, SCLC); 2) neurological or hemodynamic unstable; 3) autoimmune diseases; 4) >4 brain metastases; 5) LM in CSF or at MRI; 6) prior WBRT or SRS/SRT to the lesion to be resected	NaSRS + Tumor resection	No	1) Maximal tolerated dose at 1-month	1) Adverse events at 1-month; 2) Resection at 36-month; 3) Intracranial control at 36-month; 4) Progression free survival at 36-month; 5) LMD at 36-month; 6) Salvage therapy at 36-month
**Clump [43] (NCT02514915)**	Interventional – II Single group	University of Pittsburgh Medical Center	24	1) Age ≥18 years; 2) KPS ≥50; 3) Histological diagnosis primary tumor; 4) No contraindication MRI; 5) MRI-diagnosed ≤4 brain metastases of >1.5cm and ≤4cm diameter, ≥1 indicated for surgery; 6) estimated survival >12 weeks; 7) Willing and able to give consent and participate in all evaluations	1) Radiosensitive tumor histology (germ cell, lymphoma, multiple myeloma, leukemia); 2) >5mm midline shift, 4th ventricle compression, or signs hydrocephalus requiring emergency decompression; 3) >4 brain metastases; 4) proximity to optic apparatus or brainstem; 5) pregnancy, lactation, or not willing to undergo contraception	NaSRS + Tumor resection	No	1) Local control at 36-month	1) Overall survival at 36-month; 2) Distant failure at 3/12-month and then every 3 months up to 48-month; 3) Quality of life (FACT-BR) at 36-month
**Buchwald [44] (NCT04895592)**	Interventional – I Non-randomized, Parallel assignment	Emory University Hospital/Winship Cancer Institute	20	1) Age ≥18 years; 2) KPS ≥60; 3) Histological diagnosis primary tumor; 4) No contraindication MRI; 5) estimated survival >12 weeks; 6) Negative pregnancy test ≥7 days prior to start SRS; 7) Willing and able to give consent and participate in all evaluations	1) Receiving immunosuppressive medications; 2) pregnancy, lactation, or not willing to undergo contraception; 3) Psychological disorder, uncontrolled illness, or other reasons interfering with patient compliance	NaSRS + Steroids + Tumor resection	No	1) Adverse events of grade ≥3 at 4-month (comparing low-dose vs high-dose steroids)	1) Density of immune niche up to 24-month; 2) Local recurrence at 24-month; 3) Distant failure at 24-month; 4) Overall survival at 24-month
**Murphy [45] (NCT01891318)**	Interventional – II Single group	Case Comprehensive Cancer Center Cleveland	36	1) Age ≥18 years; 2) KPS ≥70; 3) No contraindication MRI; 4) MRI-diagnosed <4 brain metastases of >2cm and ≤5cm diameter, one indicated for surgery; 5) Willing and able to give consent and participate in all evaluations	1) Radiosensitive tumor histology (germ cell, lymphoma, SCLC); 2) >4 brain metastases; 3) LM in CSF or at MRI; 4) prior WBRT; 5) proximity to optic chiasm and/or brainstem not allowing to deliver 10 Gy dose; 6) Psychological disorder, illness, or other reasons interfering with patient compliance	NaSRS + Tumor resection	No	1) Maximal tolerated dose at 0-days; 2) Local control at 36-month	1) Distant control at 36-month; 2) Radiation necrosis and steroid dependency at 36-month; 3) Salvage therapy at 36-month

**Yan [46] (NCT03750227)**	Interventional – II Randomized, Parallel assignment	Mayo Clinic	140	1) Age ≥18 years; 2) ECOG ≤2; 3) Histological diagnosis primary tumor; 4) MRI-diagnosed ≤10 brain metastases of ≤5cm diameter, one indicated for surgery; 5) Willing and able to give consent and participate in all evaluations	1) Radiosensitive tumor histology (germ cell, lymphoma, multiple myeloma, leukemia); 2) Brain metastases located ≤5mm off the optic chiasm; 4) >10 brain metastases and/or of ≥5cm diameter; 5) LM in CSF or at MRI; 6) prior WBRT and/or brain surgery; 7) surgical indication of ≥2 brain metastases; 8) Indication for ≥4 weeks of steroids or bevacizumab; 9) pregnancy, lactation, or not willing to undergo contraception; 10) Psychological disorder, unstable illness, or other reasons interfering with patient compliance	NaSRS + Tumor resection	Tumor resection + Postoperative SRS	1) CNS composite endpoint event at 60-month	1) Overall survival at 60-month; 2) Adverse event at 60-month; 3) CNS composite endpoint event adjusted survival at 60-month; 4) CNS composite endpoint event free event rate at 60-month; 5) Quality of life (FACT-BR) at 6-month; 6) Rate completion therapy up to 60-month; 7) Time systemic therapy up to 60-month; 8) Time regional progression up to 60-month; 9) Time CNS progression up to 60-month; 10) Time subsequent therapy including WBRT up to 60-month; 11) Rate neurosurgery morbidity up to 60-month
**Faruqi and Patel [36] (NCT04474925)**	Interventional – II Randomized, Parallel assignment	AHS Cancer Control Alberta	88	1) Age ≥18 years; 2) ECOG 0-2; 3) Histological diagnosis primary tumor; 4) Ability to complete neurocognitive assessment independently; 5) Negative pregnancy test ≥7 days prior to start SRS; 6) Willing and able to give consent and participate in all evaluations	1) Radiosensitive tumor histology (germ cell, SCLC, hematologic malignancies); 2) LM in CSF or at MRI; 3) prior WBRT or SRS/SRT to the lesion to be resected; 4) unable to undergo MRI	NaSRS + Tumor resection	Tumor resection + Postoperative SRS	1) Local control at 12-month	1) Local control at 6/24-month; 2) Distant failure at 6/12/24-month; 3) LM at 6/12/24-month; 4) Overall survival at 6/12/24-month; 5) Hopkins verbal learning at 3/6/9/12/16/24-month; 6) Controlled oral word association at 3/6/9/12/16/24-month; 7) Trial Making test at 3/6/9/12/16/24-month
**Yeboa [37] (NCT03741673)**	Interventional – II Randomized, Parallel assignment	M.D. Anderson Cancer Center	86	1) Age ≥18 years; 2) KPS ≥70 and ECOG ≥2; 3) Histological diagnosis primary tumor; 4) No contraindication MRI; 5) MRI-diagnosed brain metastases of ≤4cm diameter for single fraction or ≤7cm for multifraction; 6) Willing and able to give consent and participate in all evaluations	1) Radiosensitive tumor histology (SCLC, germ cell, lymphoma, multiple myeloma, leukemia); 2) LM in CSF or at MRI ;3) pregnancy, lactation, or not willing to undergo contraception	NaSRS + Tumor resection	Tumor resection + Postoperative SRS	1) LM-free at 12-month	1) Local control at 12-month; 2) Distant control at 12-month; 3) Delta radiomics up to 48-month; 4) Circulating tumor cells up to 48-month; 5) CSF analysis up to 48-month; 6) Cognitive function (HVLT-R, COWA, TMT, CTB-COMP) up to 12-month; 7) Symptom burden questionnaire

									(MDASI-BT) up to 48-month; 8) Quality of life (EQ-5D-5L) at 12-month
**Wu [38] (NCT05267587)**	Interventional – II Single group	H. Lee Moffitt Cancer Center and Research Institute	60	1) Age ≥18 years; 2) KPS ≥60; 3) No contraindication MRI; 4) MRI-diagnosed ≥1 brain metastases of >1cm and ≤6cm diameter, one indicated for surgery; 5) estimated survival >3 months; 6) Negative pregnancy test ≥7 days prior to start SRS; 7) Willing and able to give consent and participate in all evaluations	11) Radiosensitive tumor histology (germ cell, lymphoma, multiple myeloma, leukemia); 2) >10mm midline shift, 4th ventricle compression, signs intracranial hypertension requiring emergency decompression; 3) Brain metastases located ≤2mm off the optic chiasm or in the brainstem; 4) LM in CSF or at MRI; 5) prior WBRT or SRS/SRT to the lesion to be resected; 6) Prior chemotherapy ≤7 days; 7) pregnancy, lactation, or not willing to undergo contraception; 9) Psychological disorder, unstable illness, autoimmunity, or other reasons interfering with patient compliance	HfNaSRS + Tumor resection	No	1) Time to progression up to 12-month	1) Time to death up to 12-month; 2) Time to LMD up to 12-month; 3) Local control at 6/12-month; 3) Distant failure at 6/12-month; 4) LM at 6/12-month
