# Supplementary material for: Neoadjuvant Stereotactic Radiotherapy for Brain Metastases: Systematic Review and Meta-Analysis of the Literature and Ongoing Clinical Trials
Source: Cancers (Basel). 2022 Sep 4;14(17):4328. doi: 10.3390/cancers14174328 (PMC9455064; doi:10.3390/cancers14174328)
Supplement: Supplementary file 1 [file cancers-14-04328-s001.zip › Supplementary File S3 - Funnel Plots.pdf]

Supplementary File S3. Funnel plots of pooled rates of: **A**. radiation necrosis; **B**. symptomatic radiation necrosis; **C**. local recurrences; **D**. distant brain recurrences; **E.** leptomeningeal disease; **F**. local control at 12 months; **G**. overall survival at 6 months; **H**. overall survival at 12 months; **I**. overall survival at 24 months.

A

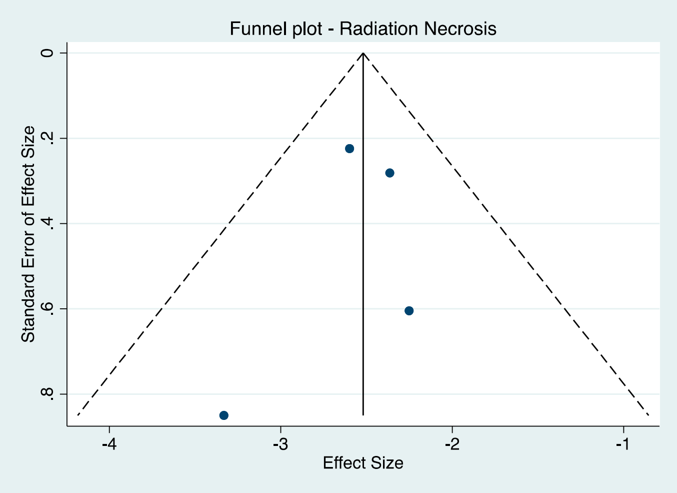

B

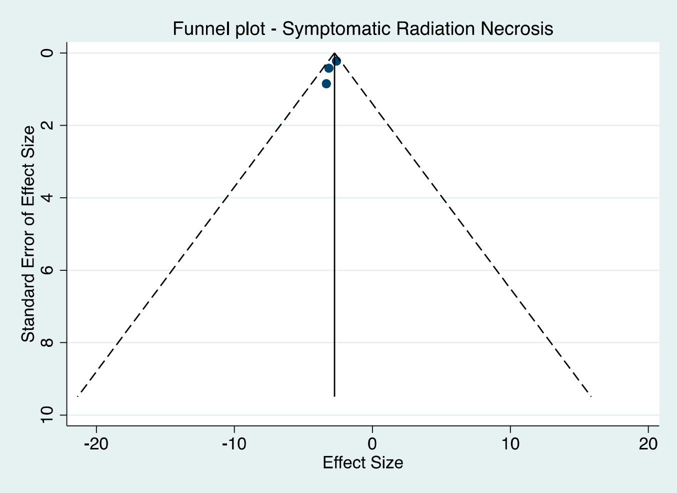

C

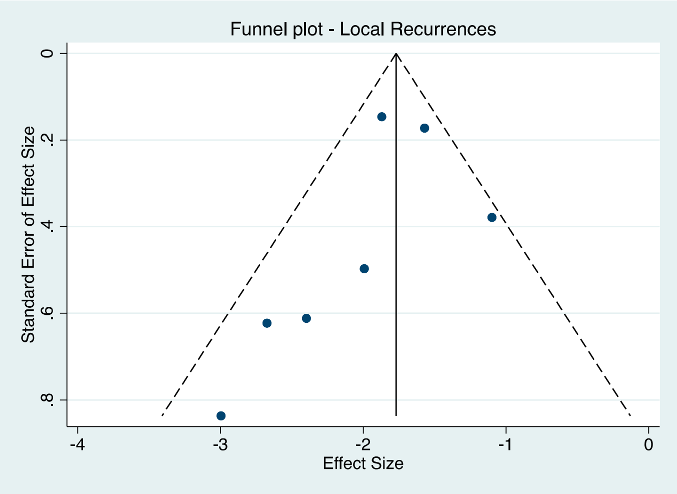

D

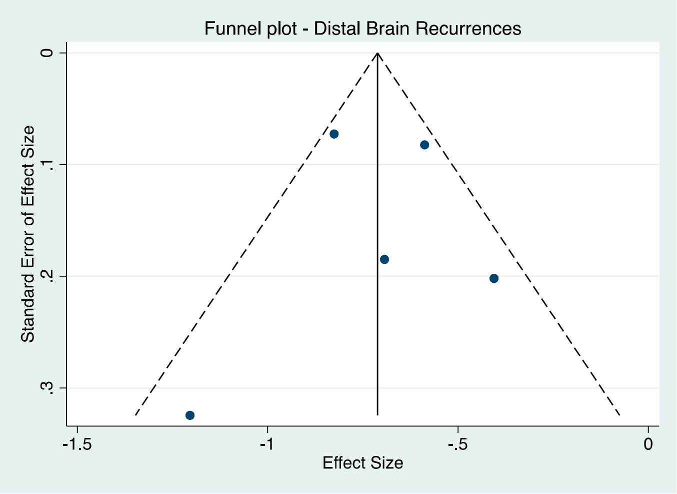

E

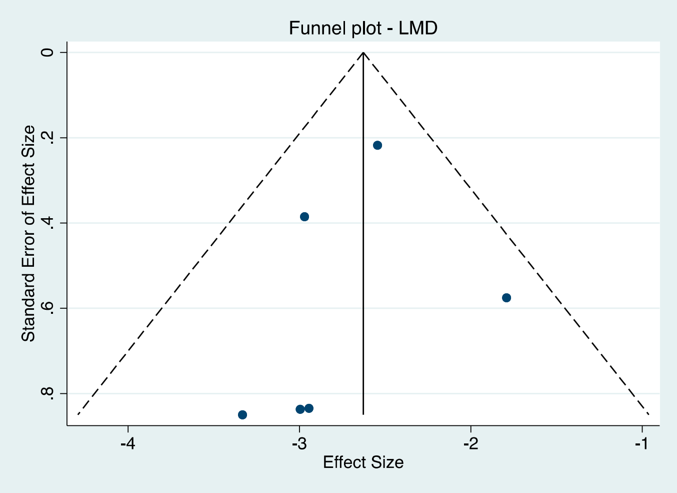

F

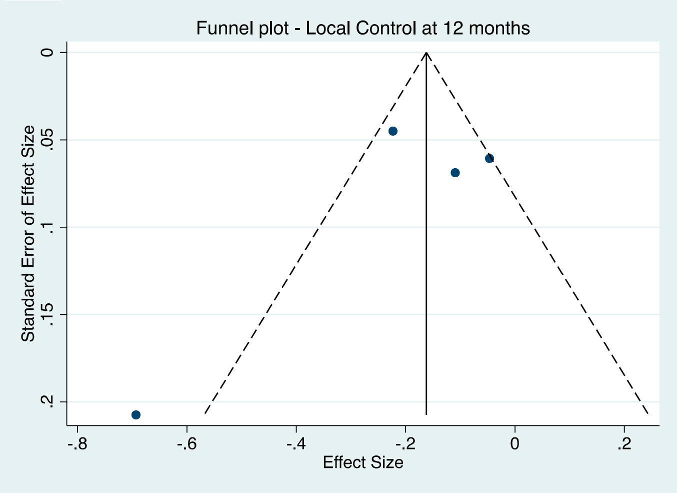

G

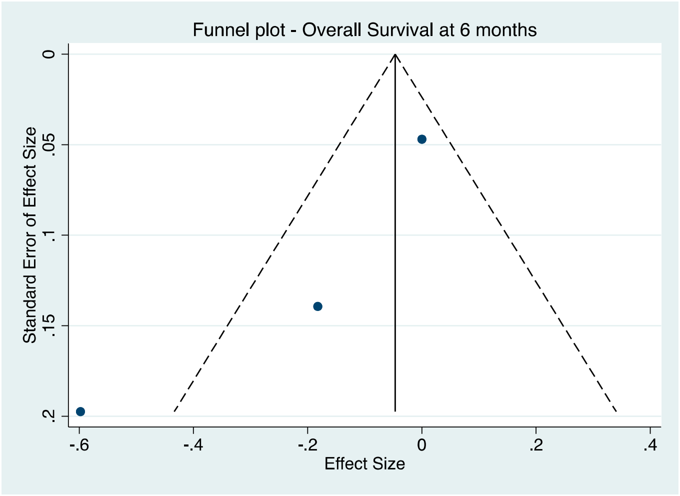
Funnel plot - Overall Survival at 6 months
Standard Error of Effect Size
0
.05
.1
.15
.2
-.6
-.4
-.2
0
.2
.4
Effect Size

H

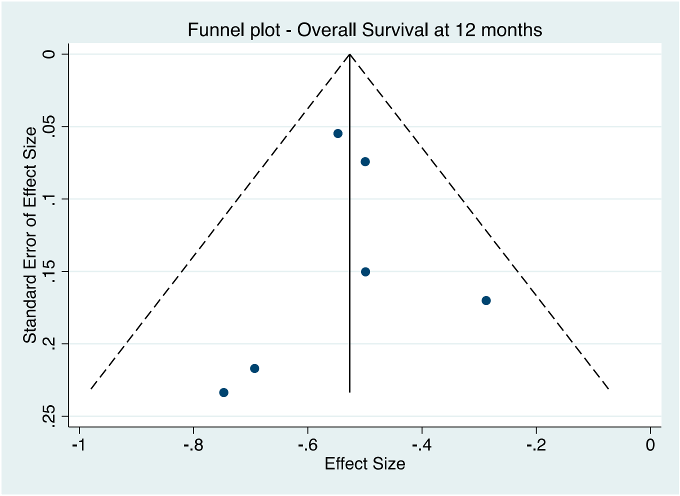
Funnel plot - Overall Survival at 12 months
Standard Error of Effect Size
0
.05
.1
.15
.2
.25
-1
-.8
-.6
-.4
-.2
0
Effect Size

I

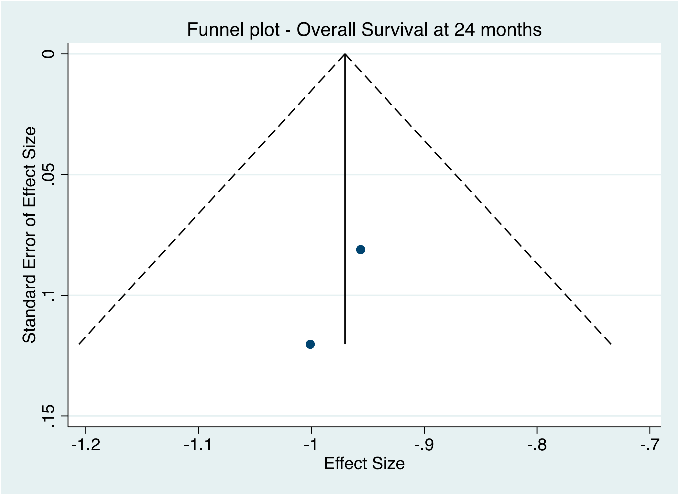
Funnel plot - Overall Survival at 24 months
Standard Error of Effect Size
0
.05
.1
.15
-1.2
-1.1
-1
-.9
-.8
-.7
Effect Size
